# A Comparison of Vasopressin, Terlipressin, and Lactated Ringers for Resuscitation of Uncontrolled Hemorrhagic Shock in an Animal Model

**DOI:** 10.1371/journal.pone.0095821

**Published:** 2014-04-23

**Authors:** Chien-Chang Lee, Meng-Tse Gabriel Lee, Shy-Shin Chang, Si-Huei Lee, Yu-Chi Huang, Chia-Hung Yo, Shih-Hao Lee, Shyr-Chyr Chen

**Affiliations:** 1 Department of Emergency Medicine, National Taiwan University Hospital, Taipei, Taiwan; 2 Graduate Institute of Clinical Medical Sciences, Chang Gung University, Taoyuan, Taiwan; 3 Department of Family Medicine and Emergency Medicine, Chang Gung Memorial Hospital, Taoyuan, Taiwan; 4 Department of Rehabilitation and Physical Medicine, National Yang-Ming University and Taipei Veterans General Hospital, National Yang-Ming University, Taipei, Taiwan; 5 Center for Geriatrics and Gerontology, Taipei Veterans General Hospital, National Yang-Ming University, Taipei, Taiwan; 6 Department of Emergency Medicine, Far Eastern Memorial Hospital, Taipei, Taiwan; University of Giessen Lung Center, Germany

## Abstract

**Aim:**

The aim of this study is to compare the effect of lactated ringer (LR), vasopressin (Vaso) or terlipressin (Terli) on uncontrolled hemorrhagic shock (UHS) in rats.

**Methods:**

48 rats were divided into four treatment groups for UHS study. Vaso group was given bolus vasopressin (0.8 U/kg); the Terli group was given bolus terlipressin (15 mcg/kg); LR group was given LR and the sham group was not given anything. Mean arterial pressure (MAP), serum lactate level, plasma cytokine levels, lung injury and mortality are investigated for these different treatment groups.

**Results:**

Compared with LR group, vasopressin and terlipressin-treated groups were associated with higher MAP, lowered mortality rates, less lung injury, lowered serum lactate level, less proinflammatory and more anti-inflammatory cytokine production at certain time points. Comparing between vasopressin and terlipressin treated groups, there is no statistical difference in mortality rates, lung injury, serum lactate level and cytokine level. However, there is a difference in the length of time in maintaining a restored level of MAP (80 to 110 mmHg). The terlipressin treated rats can maintain this restored level of MAP for 45 minutes, but the vasopressin treated rats can only maintain this restored level of MAP for 5 minutes before decreasing gradually to the MAP observed in LR group (40 mmHg).

**Conclusion:**

Early optimization of hemodynamics with terlipressin or vasopressin in an animal model of UHS was associated with improved hemodynamics and inflammatory cytokine profile than the LR control. Compared with vasopressin, terlipressin has the advantage of ease of use and sustained effects.

## Introduction

Fluid resuscitation is a standard therapy for uncontrolled hemorrhage shock (UHS) but deciding when is the correct timing for fluid resuscitation is challenging. Both animal and clinical studies have revealed that attempting to achieve normal blood pressure by aggressive fluid resuscitation during uncontrolled hemorrhagic shock (UHS) increased mortality [1]–[5]. Early aggressive fluid resuscitation in the presence of uncontrolled hemorrhage usually induces further blood loss, worsens acidosis, results in severe hemodilution, decreases oxygen delivery, and increases mortality [2]–[5]. However, there is a risk that a delay in fluid resuscitation might increase the likelihood of organ damage from impaired perfusion [6], [7]. Furthermore, hemorrhagic shock of marked duration may progress to cardiovascular collapse that is unresponsive to volume replacement and catecholamine infusion. Therefore, using adjunct vasopressors to raise blood pressure in the early course of hemorrhagic shock, appears as a reasonable strategy.

Using a vasopressor early may avoid the detrimental effects associated with aggressive fluid resuscitation, while maintaining a level of adequate tissue perfusion for a short period of time. Vasopressin is a natural nonapeptide hormone synthesized in the hypothalamus that can increase blood pressure via vasoconstriction. Deficiency of vasopressin can contribute to hypotension observed in patients with catecholamine-resistant septic shock, postcardiotomy shock and cardiac arrest [8]. The short-term survival benefits of vasopressin in hemorrhagic shock have been shown in both animal and human studies [9]–[16]. However, we are not aware of any human studies that use vasopressin in the early phases of resuscitation from hemorrhagic shock.

Clinical administration of vasopressin for patients with refractive shock may require continuous infusion due to its short half-life of 10∼35 minutes. This may preclude their use in the pre-hospital setting where severe hemorrhagic shock occurs. Terlipressin is an analogue of vasopressin with a longer half-life, and is widely applied in the treatment of esophageal varices bleeding. It can be given by bolus injection, which may be a more practical method of drug administration in the prehospital setting. However, the role of terlipressin in traumatic shock has not been widely studied and to our knowledge there is only one published report on this topic [17].

In this study, we attempted to compare the effects on inflammatory response or acute lung injury with early use of vasopressin, terlipressin or LR in an animal model of profound hemorrhagic shock. We hypothesized that vasopressor administration may shorten the shock period and therefore decrease the acute lung injury and resultant release of pro-inflammatory cytokines.

## Methods

### Ethics Statement

All rats were maintained in accordance with the recommendations of the Guide for the Care and Use of Laboratory Animals. The National Taiwan University Hospital Animal Protocol Review Committee approved all animal protocols (approval ID: 20060318).

### Experimental Animals and MAP determination

Before inducing hemorrhagic shock, the rats were fasted overnight but allowed water ad libitum. Wistar male rats (n = 48; 12/group; weight 300–400 g) were anesthetized with intraperitoneal injections of sodium pentobarbital (50 mg/kg) and this was maintained with supplemental doses (5 mg) of pentobarbital throughout the 135-min observation period as needed. With an aseptic technique, the left femoral artery and vein were isolated and cannulated with polyethylene catheters (PE-50, Becton Dickinson, Sparks, MD). The arterial catheter was connected to a pressure transducer (BP-2 Digital Blood Pressure Monitor; Columbus Instruments, Columbus, Ohio). It was used for blood withdrawal and continuous hemodynamic monitoring. At each time point, the MAP is recorded once. The venous catheter was used for fluid and vasopressor infusion.

### Rat model of uncontrolled hemorrhagic shock

**Figure 1:**
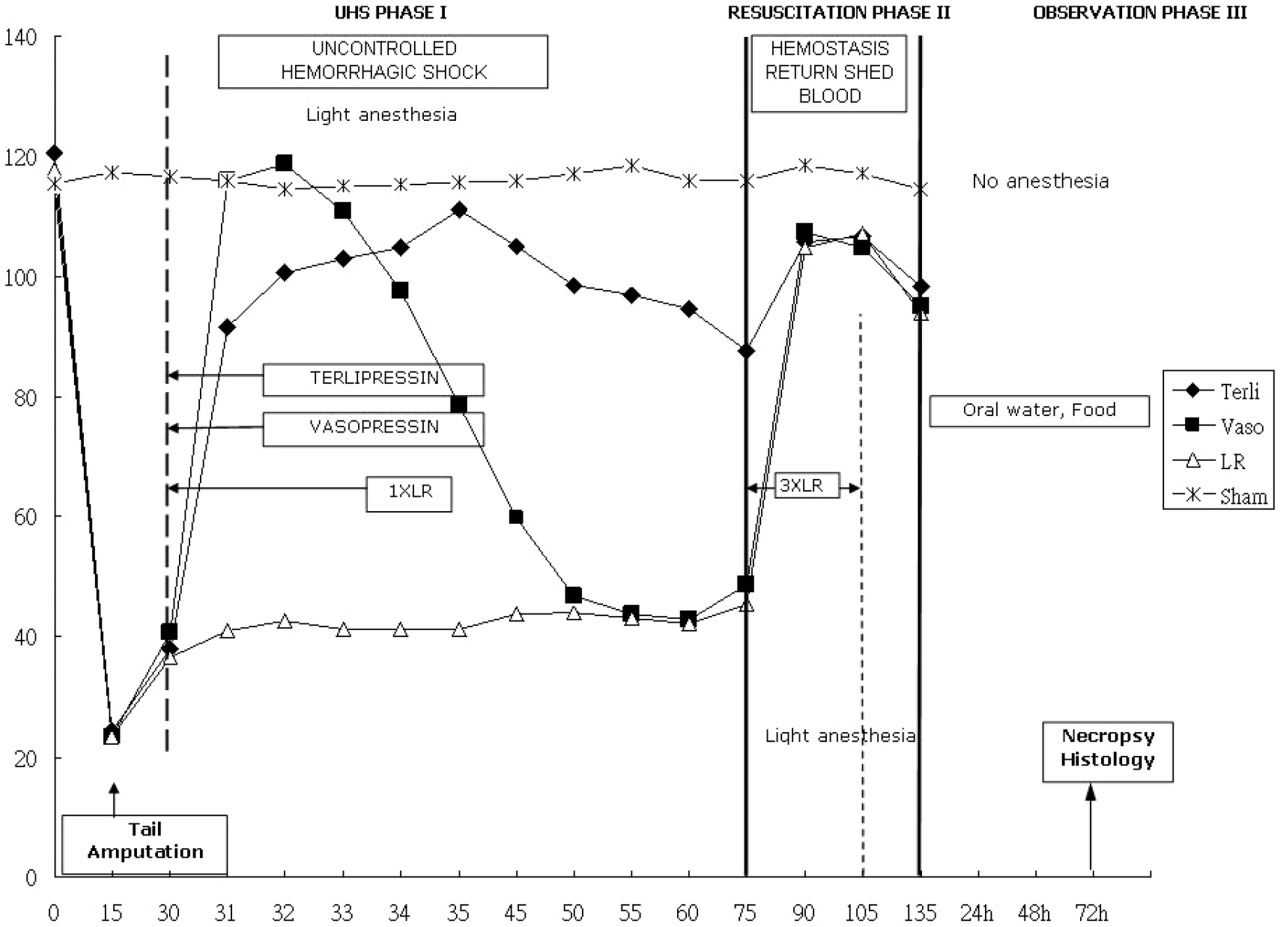
Experimental protocol and mean arterial pressure over time in four treatment groups. The MAP for the sham group is around 110

We used the modified UHS model developed by Capone and Safer et al [18]. The model was designed to simulate the life-support chain of emergency medical service for trauma, consisting of three phases (Fig 1). Phase I, UHS, (representing “prehospital” care) was from 0 to 75 minutes and included an initial 15-minute volume-controlled hemorrhage (3 mL/100 g). The same person made an effort to withdraw blood at the same speed using a syringe during volume-controlled hemorrhage. After that, uncontrolled hemorrhage was done by an 80% tail amputation. No tail blood is collected but care is taken to make sure all the 48 rats are treated in the same way. During phase I, hypotension (40 mmHg) was continued until the animals could not maintain this pressure unless extra lactated Ringer's solution (LR) was given. Phase II, resuscitation, (representing “hospital” care) spanned 60 minutes, and took place from 75 minutes to 135 minutes. It included hemostasis and infusion of a 3X the volume of shed blood solution that is made up by lactated Ringer's solution. Shed blood and lactated Ringer's solution are mixed before infusing to restore normotension. Phase III, observation, lasting from 135 mins to 72 hrs, included outcome evaluation in terms of survival and histopathology of lungs. Forty-eight animals were randomly assigned to four treatment groups. During phase I, the Vaso group animals (n = 12) received intravenous 0.8 U/kg vasopressin (Pitressin, Parke-Davis) bolus 30 min after the start of blood withdrawl (mean arterial pressure  = 40 mmHg). The use of 0.8 U/kg vasopressin is guided by a report that 0.8 U/kg Vasopressin can increase MAP in rats [19]. The Terli group animals (n = 12) received intravenous 15 µ/kg terlipressin (Glypressin, Ferring) bolus 30 min after the start of blood withdrawl. The use of 15 µ/kg terlipressin is guided by a report that <6 µ/kg and >20 µ/kg of terlipressin is needed to increase MAP in rats that undergo surgical operation [20]. The LR group animals (n = 12) received an equal volume of shed-blood lactated Ringer solution 30 min after the start of blood withdrawal and did not receive any vasopressors. The Sham group animals underwent exactly the same anesthetic regimen and monitoring as the study animals but without hemorrhagic shock, tail amputation, and fluid resuscitation. During phase II, or the period from 75 to 105 minutes after the start of the experiment, the shed blood was returned. This consisted of fluid resuscitation in the form of shed blood plus 2X LR. Hence, the total infused volume is 3X the volume of shed blood. During phases I and II, the rats were under light general anesthesia and during phase III they were awake. Light anesthesia refers to animals that are unconsciousness and immobile. As described in detail in experimental Animals, rats are first anesthetized by sodium pentobarbital (50 mg/kg) and supplemental doses (5 mg) of pentobarbital is administrated throughout the phase II period whenever the rat make a movement. After 135-min observation period, the animals were then allowed food and water ad libitum, and were monitored for three days to record their survival. At the end of 72 hours, all animals were euthanized.

### Determination of lactate, hematocrits, and cytokines

2 ml of blood samples were obtained for hematocrits and serum levels of lactate and cytokines at 30, 60, 90, and 150 min. Rat IL-6, IL-10, and TNF-α were determined by a commercially available ELISA with specific monoclonal antibodies, performed according to the manufacturer's instructions (Biosource International, USA). The reliability of these cytokine kits has been shown in previous studies [21], [22]. The detection limit was 4 pg/mL, 8 pg/mL, and 5 pg/mL of rat TNF- α, rat IL-6, and rat IL-10, respectively.

### Pathology

After the rats were euthanized, the lungs were removed from the thorax en bloc with the heart, and all blood was removed from circulation by an infusion of lactated Ringer's solution into the main pulmonary artery. After dissection, the right lower lobes were subjected to a continuous distending pressure of 4 cm H_2_O by the injection of 10% formaldehyde. After 4 hrs, they were placed in formaldehyde for several days. Two tissue samples from the right lower lobe (approximately 20×20×4 mm) were taken from central and peripheral locations for histologic examination using hematoxylin–eosin staining. To describe the criteria of edema and overall interstitial infiltration of mononuclear inflammatory cells, the following score system was employed: 0 =  none at all, 1 =  mild, 2 =  moderate, 3 =  severe (steps of 0.5 were also used for intermediate ranking) [23]. To rule out model bias, the two investigators carrying out the histological assessment were unaware of the lung sample treatment group.

### Statistics

Descriptive values are presented as mean ± standard deviation for continuous variables and absolute count with relative frequencies for categorical variables. Unless stated otherwise, all graphical points represent a single comparison of the average over time. Differences of hemodynamic parameters and serum levels of cytokine between and within the three treatment groups of animals were analyzed by repeated measure analysis of variance (rmANOVA) with a grouping factor. The repeated measure was time, and treatment strategy was used as the grouping factor. The multivariate normality, homogeneity of covariance matrices, independence, sphericity assumptions of rmANOVA was checked to make sure they were not grossly violated. Mauchly's test was used to check for sphericity. If the sphericity assumption is not met, the Greenhouse-Geisser correction was applied to adjust the degree of freedom for the test of the interaction effect between different time point and different treatment groups. As the group by time results may be solely due to the sham group differing from the other three, we limited the comparison to the 3 treatment groups of clinical interest. Post hoc analysis was performed using Tukey's method. A nonparametric Kruskal-Wallis test with Dunn's posttest was used to evaluate differences in the histologic scores. These scores are displayed as box plots with the boxes extending from the 25th to the 75th percentile. The mean difference between indicated treatment groups was considered to be significant at 0.05.

## Results

### Mean arterial pressure (MAP)

Serial measurements of mean (±SD) arterial pressure versus time plots are shown in Fig 1 & S1. The main difference of MAP occurred in the UHS part of phase I (30∼75 min). The MAP in the Terli group rose soon after bolus injection of terlipressin and remained between 80 and 110 mmHg through UHS phase I (30∼75 min). In the Vaso group, the MAP soon rose as high as 120 mmHg in response to an intravenous injection of vasopressin, but the blood pressure could not be maintained and declined gradually to as low as the LR group after 50 min. The MAP in the LR group was the lowest among the four groups, kept around 40 mmHg throughout UHS phase I. Mauchly's test showed that the sphericity assumption had been validated. Therfore, we used Greenhouse-Geisser correction to adjust the degree of freedom in rmANOVA and test the interaction effect between groups and different time points. By rmANOVA and Greenhouse-Geisser correction, the group time interaction among the three experimental groups (excluding the Sham group) was significantly different (P<0.001). In post hoc comparisons, the Terli group had a significantly higher MAP than the LR group from 30 to 75 min (p<0.001). The Vaso group had a significantly higher MAP than the LR group from 30 to 50 min (p<0.001).

### Serum lactate levels

**Figure 2:**
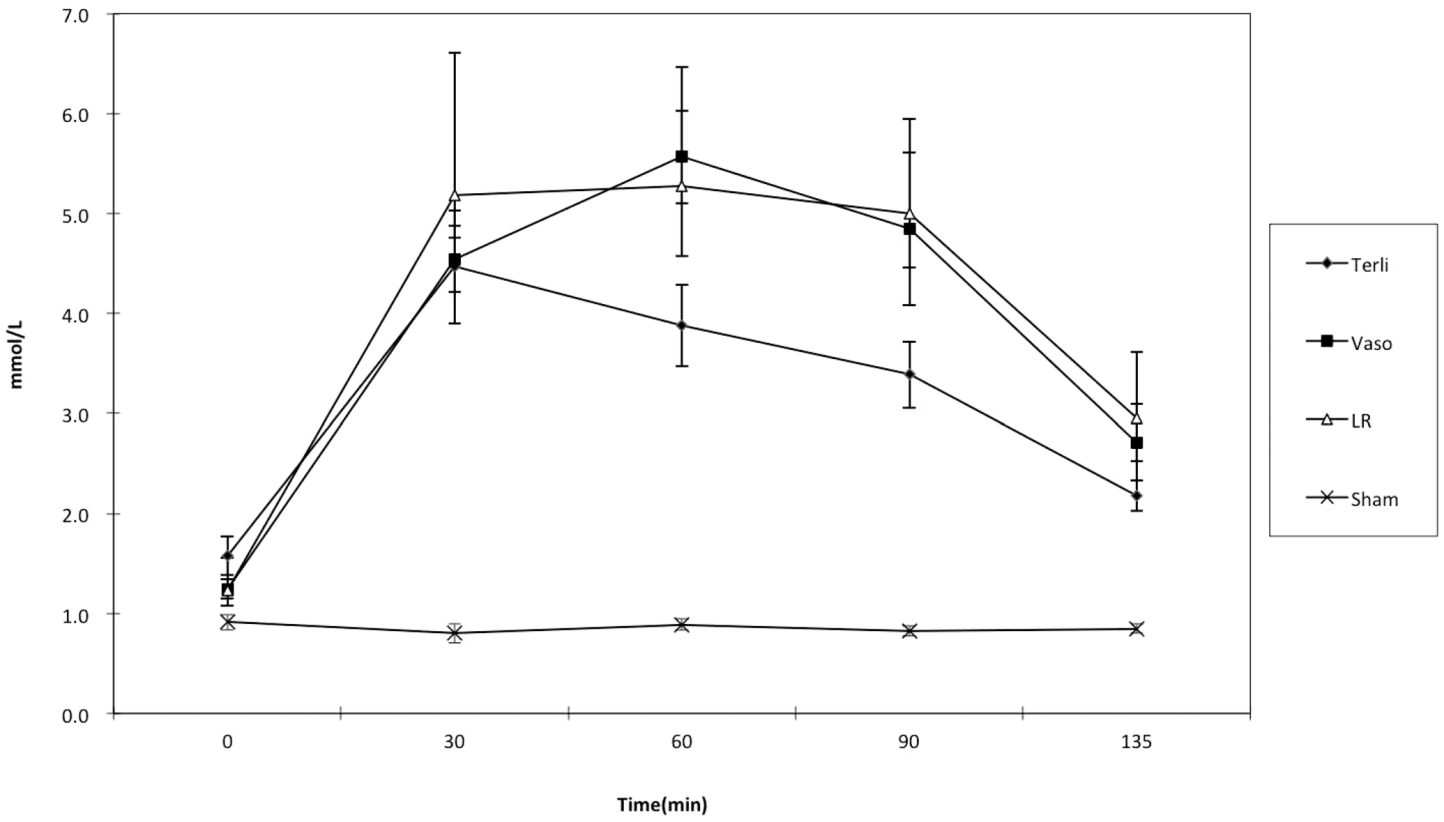
Comparison of serum lactate levels between different resuscitation strategies groups from 0 to 135 min. Data are shown as mean values and standard deviations. There were no significant differences in baseline levels. The Terli group had significantly lower serum lactate level than Vaso or LR group at 60 and 90

Serial measurements of mean (±SD) serum lactate level versus time plots in the four groups are shown in Fig 2. The serum lactate levels rose after inducing hemorrhagic shock in the first three groups of animals but not in the Sham group. In response to LR resuscitation or adjunct vasopressor in UHS phase I (30∼75 min), serum lactate decreased gradually. The sphericity assumption was not violated according to the Mauchly's test. The Terli group had nearly significant lower serum lactate levels than the LR or Vaso groups at 60 mins (p = 0.0627) and 90 mins (p = 0.089). However, after aggressive fluid resuscitation in UHS phase II (75∼105 min), there was no significant difference in serum lactate levels among the three resuscitation groups at 135 min (F statistic, p = 0.71). Sham group animals did not undergo hemorrhagic shock, and the serum lactate level remained low throughout the course of the experiment.

### Plasma cytokine levels

In all the cytokine tests, the variances for all the treatment groups vary greatly over time and the sphericity assumption is not met. Hence, Greenhouse-Geisser correction is used to adjust the degree of freedom in rmANOVA and test the interaction effect between groups and different time points.

**Figure 3:**
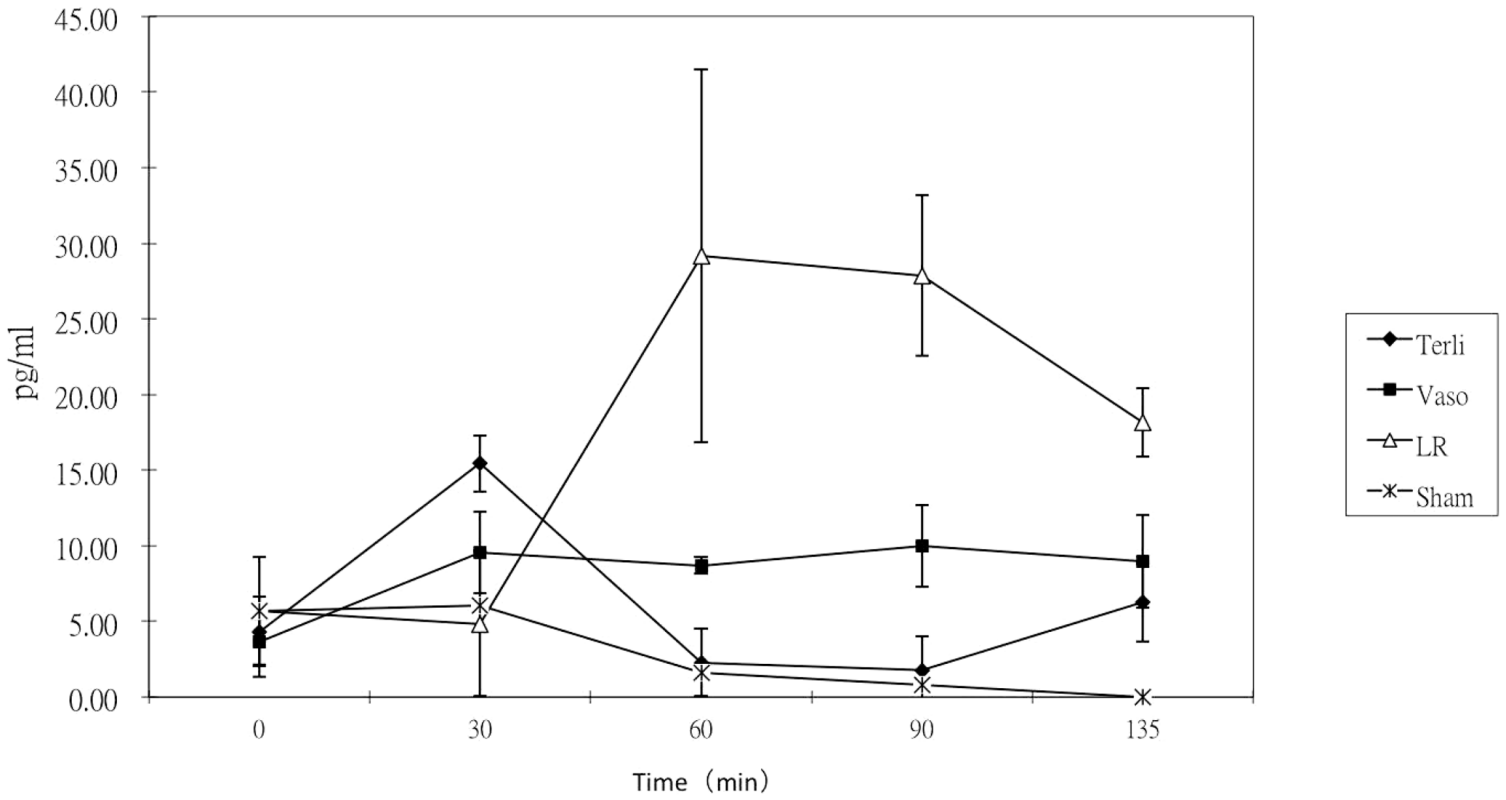
Comparison of serum IL-6 levels between different resuscitation strategies groups from 0 to 135 min. Data are shown as mean values and standard deviations. There were no significant differences in baseline levels. Serum levels of IL-6 in the LR group were significantly higher than those in the Vaso (P<0.001), and Terli (P<0.001) groups from 60 to 135 mins. Serum levels of IL-6 in the Vaso group were significantly higher than those in the Terli (P<0.001) group from 60 to 90 mins. There were no significant differences of serum IL-6 levels between the Vaso and Terli groups (p = 0.54) at the end of observation (135 min).

Serial measurements of mean (±SD) plasma IL-6 levels versus time plots are shown in Fig. 3. The IL-6 level for LR group increase rapidly between 30–60 minutes and then slowly declines. Other groups didn't show this rapid increase. By rmANOVA and Greenhouse-Geisser correction, the group time interaction among the three experimental groups (excluding the Sham group) was significantly different (P<0.001). Post hoc comparisons showed that the IL-6 level of the LR group was significantly higher than those in the Vaso (P<0.001), and Terli (P<0.001) groups for most part of the experiment, ranging from 60 to 135 mins. IL-6 level in the Vaso group were significantly higher than those in the Terli (P<0.01) group only at 60 to 90 mins.

**Figure 4:**
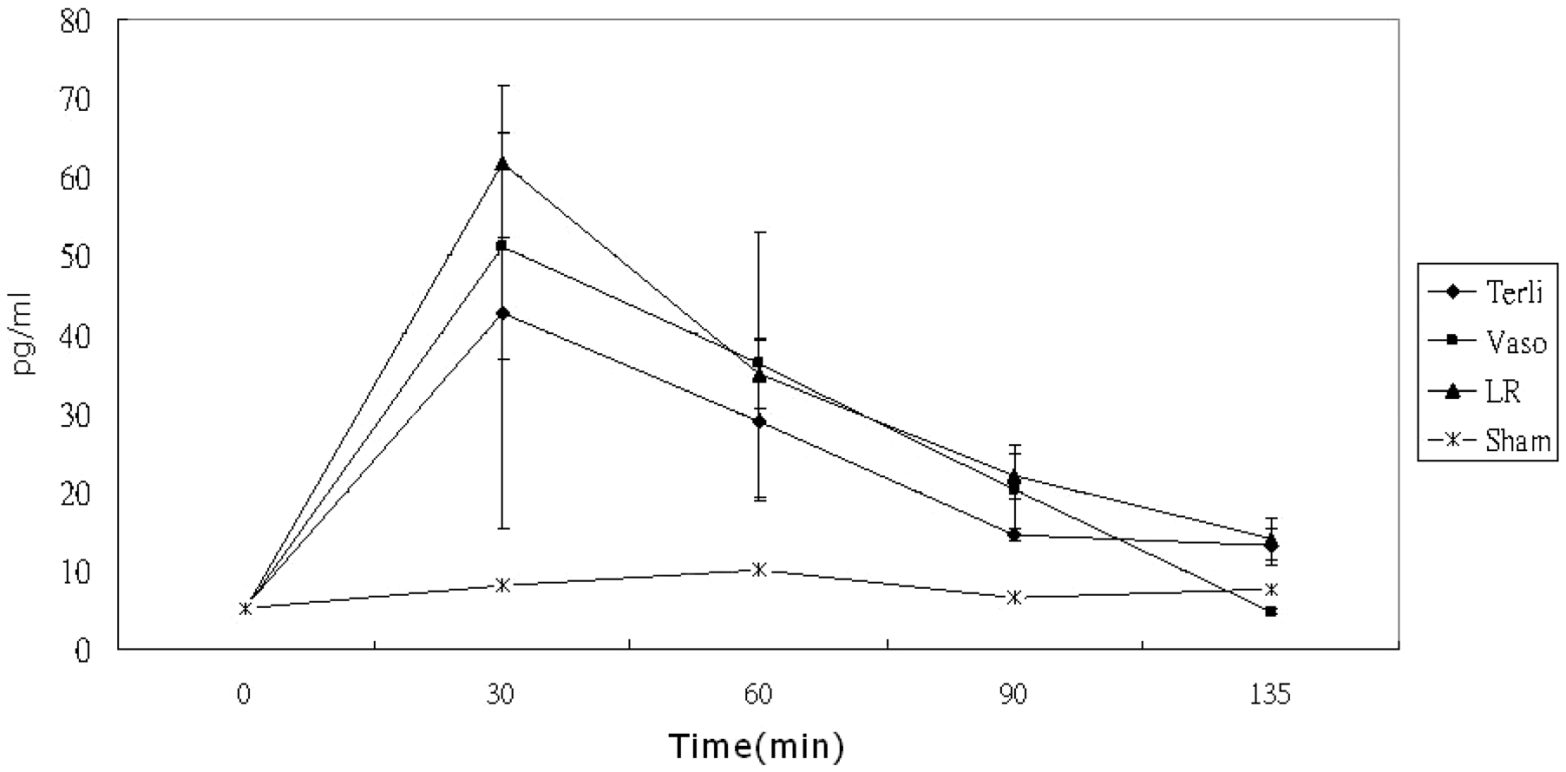
Comparison of serum TNF-alpha levels between different resuscitation strategies groups from 0 to 135 min. Data are shown as mean values and standard deviations. There were no significant differences in baseline levels and the serum TNF-alpha levels over time.

Serial measurements of mean (±SD) plasma TNF-α levels versus time plots are shown in Fig. 4. Compared with IL-6, serum concentrations of TNF-α peaked early at 30 minutes and declined rapidly, while IL-6 peaked later at 60 to 135 minutes. By rmANOVA and Greenhouse-Geisser correction, the group time interaction among the three experimental groups (excluding the Sham group) was not significantly different (P = 0.10). A post hoc Tukey test showed that all pairwise comparisons were insignificant at α = 0.01.

**Figure 5:**
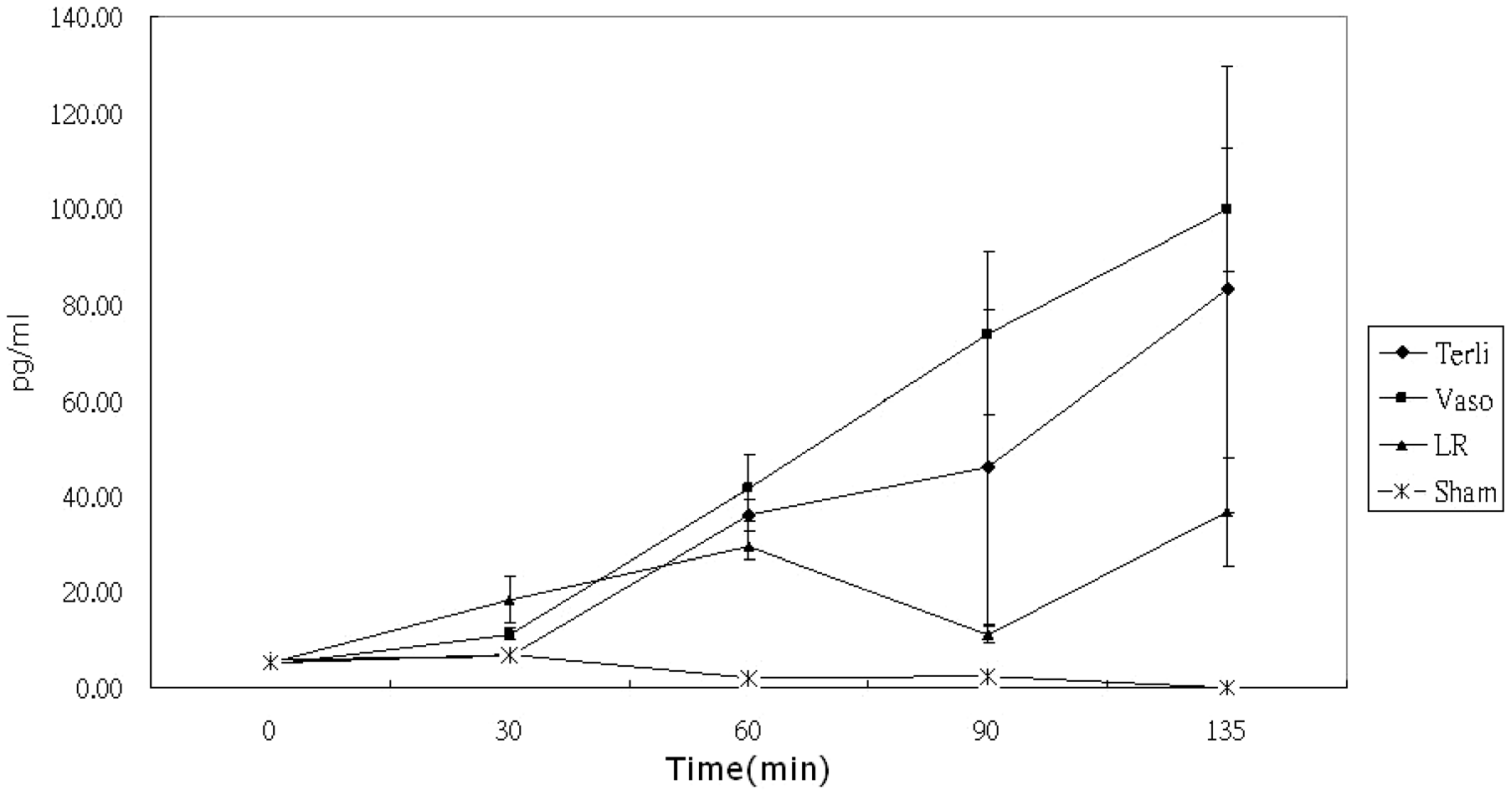
Comparison of serum IL-10 levels between different resuscitation strategies groups from 0 to 135 min. Data are shown as mean values and standard deviations. There were no significant differences in baseline levels. IL-10 production in the Terli and Vaso groups increased more significantly than in the LR or Sham groups (P<0.001) but there was no significant difference of serum levels of IL-10 between the Terli and Vaso groups.

Serial measurements of mean (±SD) plasma IL-10 levels versus time plots are shown in Fig. 5. By rmANOVA and Greenhouse-Geisser correction, the group time interaction was significant among the three groups (P<0.0086). A post hoc Tukey test showed that IL-10 levels in the Vaso group were significantly higher than those in the LR (P<0.01) group only at 90 mins.

### Histopathology

**Figure 6:**
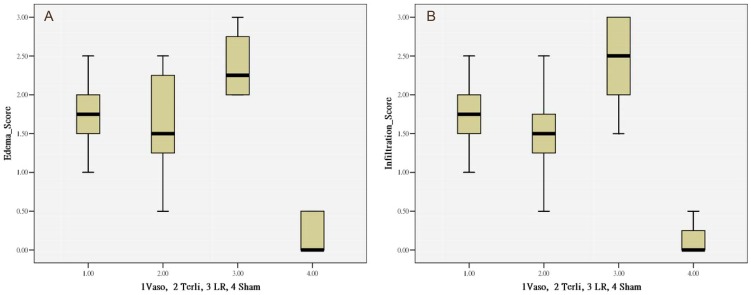
Histologic variables with regard to pulmonary edema formation and interstitial cell infiltration. *A*, Edema score, significant comparisons: Terli vs. LR (p = 0.027) or Vaso vs. LR (p = 0.01). *B*, Cell infiltration score, significant comparisons Terli vs. LR (p = 0.020) or Vaso vs. LR (p = 0.031). The Sham group showed significant lower pathologic scores than all other resuscitation groups (*P<0.001*).

The histopathology changes due to shock injury were observed in the lung tissue. Both edema and infiltration scores showed a trend toward milder histopathologic changes in the adjunct vasopressor groups as compared to the LR group. Figure 6 shows histologic variables with significant differences between the groups with regard to pulmonary edema formation and interstitial cell infiltration. Significant pair-wise comparisons regarding edema scores in the three resuscitation groups were noted in Terli vs. LR (p = 0.027) or Vaso vs. LR (p = 0.01). Significant pair-wise comparisons regarding infiltration scores were noted in Terli vs. LR (p = 0.020) or Vaso vs. LR (p = 0.031). The Sham group showed significantly lower pathologic scores than all other resuscitation groups (P<0.001). There was no significant difference in histopathologic changes between animals resuscitated with either vasopressin or terlipressin.

### Mortality

It is found that the experiment resulted in 10 deaths during the 72 hours of study, with three (25%) in the Terli group, three (25%) in the Vaso group, four (33.3%) in the LR group, and none in the Sham group. Data on the non-surviving animals during the experiment are not included in any analysis except for mortality count at the end of 72 hours. There was no significant difference in mortality between Terli, Vaso, or LR groups.

## Discussion

Compared with vasopressin or standard LR resuscitation, our study showed superior effects of terlipressin on MAP in this animal model of uncontrolled hemorrhagic shock. The use of adjunct vasopressor, vasopressin or terlipressin was associated with less proinflammatory cytokine production and less postresuscitation lung injury compared with standard LR resuscitation alone. We were not able to detect any statistical difference in mortality among the three groups. Comparing the two vasopressors, our study did not show any significant difference with respect to cytokine profile and histologic changes between surviving animals resuscitated with terlipressin or vasopressin.

Despite the promising results, the clinical use of vasopressin in the prehospital setting may be hampered by its short half-life and the requirement for continuous infusion. A recent ongoing multicenter trial (VITRIS-Study) assessing the effect of vasopressin on pre-hospital shock patients designed three times bolus injection for 15 minutes depending on the patient's response [24]. However, this simplified way of vasopressin administration might cause delay for pre-hospital resuscitation. Furthermore, the effect of vasopressin is not likely to sustain for too long [24]. Terlipressin is a longer-acting agent that can be given in just one bolus, and therefore is in our opinion more suitable for use in patients that require long traveling time to the trauma center such as those on desolate battlefield.

Terlipressin is a synthetic prodrug that is converted to lysine vasopressin and results in a rapid and sustained release of the structural analog lysine vasopressin. Both vasopressin and it's analog, through the V1-receptor stimulation, produces more potent systemic and mesenteric vasoconstriction than either angiotensin II or norepinephrine and potentiated responses to norepinephrine [25], [26]. The molecular mechanism on how Vasopressin and it's analog increase vasoconstriction is unclear, but ongoing research suggest that many signal transduction proteins such as Phospholipase C (PLC), protein kinase C (PKC), myosin light chain phosphatase (MLCP), and myosin light chain (MLC20) are involved [27].

Terlipressin is widely used in the treatment of acute bleeding from gastric or esophageal varices [25]. In a systemic review, terlipressin is the only drug with proven efficacy in reducing mortality from variceal bleeding [28]. Besides, the use of terlipressin was not restricted in variceal bleeding. It has been shown to reduce blood loss without impairment in wound healing during wound excision and skin grafting in burn patients [29]. Therefore, we thought terlipressin might be a reasonable choice of adjunct vasopressors in the management of trauma related UHS.

In addition to the hemodynamic effect, our study also showed that the use of adjunct vasopressors is associated with less lung injury and a favored inflammatory cytokine profile. Compared with standard LR-resuscitated animals, both terlipressin and vasopressin-treated animals showed reduced production of proinflammatory IL-6, and increased production of anti-inflammatory IL-10. Prolonged hemorrhagic shock may induce global tissue hypoxia, which in itself is a stimulus of systemic inflammation. Besides, restoration of circulation after prolonged shock is associated with another distinct form of organ injury, ischemia-reperfusion injury. This could be directly attributable to the increased production of toxic reactive oxygen intermediates, increased production of inflammatory mediators, and activation of pro-apoptotic pathways [21], [30]–[32]. The reactive oxygen species and inflammatory mediators have been shown to be associated with post trauma-resuscitation acute lung injury, myocardial dysfunction, and even multiple organ failure syndrome [30], [31], [33]–[36]. In addition, it is also shown that in brain dead rats that hemodynamic resuscitation with arginine vasopressin decrease pro-inflammatory cytokines in serum [37]. Despite the different causes of shock, our experiments agreed with other findings that improving hemodynamics in the early phase of shock is associated with a favored profile of inflammatory cytokine production. The molecular mechanism on how vasopressors can reduce proinflammatory cytokine production is unclear but recent studies using V2 G protein-coupled receptor antagonist followed by immunoblotting suggest that the proinflammatory effect might be related to downstream signaling via V2 receptor and NF-κB [38].

Although there was a trend toward better inflammatory cytokine profiles and histopathological change in terlipressin-treated rats, we could not demonstrate a statistically significant difference between rats treated with vasopressin or terlipressin. Several limitations to this study should be noted. First, it is technically difficult to have a super large animal sample size to identify mortality differences between groups. At a one-sided alpha level of 0.05 and a beta level of 0.2, at least 279 animals in each group are required to identify a mortality difference of 10%. We were unable to do an animal research at this scale. In addition, all the surviving rats are euthanized at day 3. The mortality rate might be different if we continue to monitor the rats for a longer period of time. Both piece of information could be useful in explaining why we see no mortality differences between the groups after 3 days. Secondly, we did not measure cerebral or intestinal blood flow, and cannot comment on the effects of terlipressin or vasopressin on cerebral blood flow throughout the experiment. Concerns on impairment of cerebral blood flow were based on poor neurologic outcomes in a previous study comparing vasopressin with epinephrine in cardiopulmonary arrest [39]. Finally, the laboratory setting did not allow for a post-resuscitational intensive care recovery, long-term survival data and if the surviving animals showed differences in neurological and behavioral outcome. Future experiments that can obtain these information would be of clinical significance.

In conclusion, our study showed that terlipressin might offer a potential therapeutic alternative in hypotensive trauma patients. Unlike vasopressin or norepinephrine that requires continuous infusion, terlipressin could be given in bolus injection, enabling its use in the prehospital setting. Our study also showed that early optimization of hemodynamics either by vasopressin or terlipressin was associated with a better inflammatory cytokine profile and lung histopathology. It would be worthwhile to carry out a trial involving larger number of animals to verify the effects observed in our study.

## Supporting Information

Figure S1 **Comparison of mean arterial pressure patterns between different resuscitation strategies groups from 0 to 135 min.** Data are shown as mean values and standard deviations. A non-linear timeline is used to find out when the MAP changes after resuscitation. There were no significant differences in baseline levels. The Terli group had significantly higher mean arterial pressure than LR group from 30 to 75 min. The Vaso group had significantly higher mean arterial pressure than LR group from 30 to 50 min.
(DOC)
