# Supplementary material for: A Comparison of Vasopressin, Terlipressin, and Lactated Ringers for Resuscitation of Uncontrolled Hemorrhagic Shock in an Animal Model
Source: PLoS One. 2014 Apr 23;9(4):e95821. doi: 10.1371/journal.pone.0095821 (PMC3997410; doi:10.1371/journal.pone.0095821)
Supplement: Figure S1 — Comparison of mean arterial pressure patterns between different resuscitation strategies groups from 0 to 135 min. Data are shown as mean values and standard deviations. A non-linear timeline is used to find out when the MAP changes after resuscitation. There were no significant differences in baseline levels. The Terli group had significantly higher mean arterial pressure than LR group from 30 to 75 min. The Vaso group had significantly higher mean arterial pressure than LR group from 30 to 50 min. (DOC) [file pone.0095821.s001.doc]

**
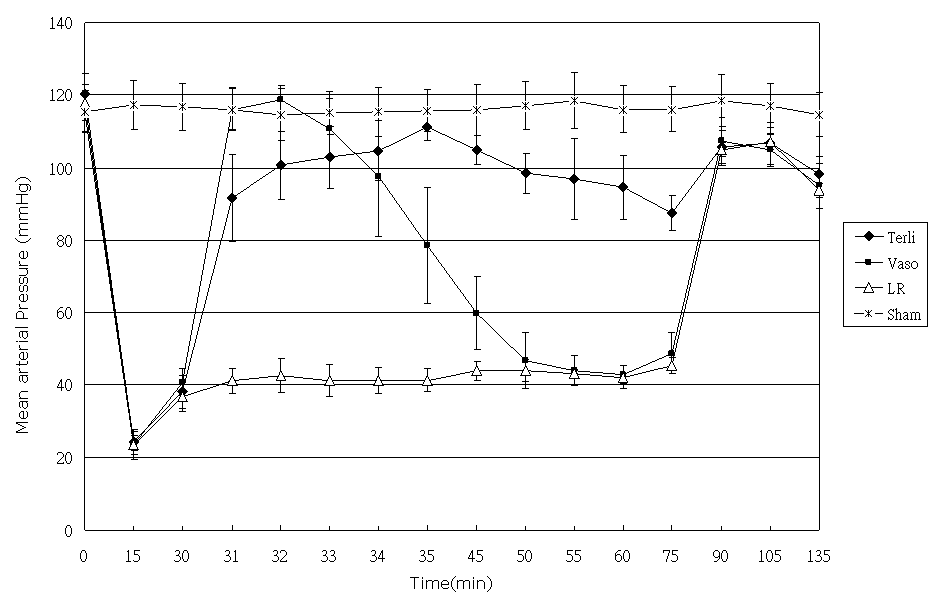
**

**Supplementary Fig 1**

Comparison of mean arterial pressure patterns between different resuscitation strategies groups from 0 to 135 min. Data are shown as mean values and standard deviations. A non-linear timeline is used to find out when the MAP changes after resuscitation. There were no significant differences in baseline levels. The Terli group had significantly higher mean arterial pressure than LR group from 30 to 75 min. The Vaso group had significantly higher mean arterial pressure than LR group from 30 to 50 min.
